# ZBTB20 Positively Regulates Oxidative Stress, Mitochondrial Fission, and Inflammatory Responses of ox-LDL-Induced Macrophages in Atherosclerosis

**DOI:** 10.1155/2021/5590855

**Published:** 2021-03-09

**Authors:** Jun Tao, Junxiong Qiu, Liuyi Lu, Lisui Zhang, Yuan Fu, Meng Wang, Jingjun Han, Maomao Shi, Ling Li, Zongkai Zhao, Feng Wei, Chao Wang, Haifeng Zhang, Shi Liang, Junmeng Zheng

**Affiliations:** ^1^Guangdong Provincial Key Laboratory of Malignant Tumor Epigenetics and Gene Regulation, Sun Yat-sen Memorial Hospital, Sun Yat-sen University, Guangzhou, China 510120; ^2^Department of Cardiovascular Surgery, Sun Yat-sen Memorial Hospital, Sun Yat-sen University, Guangzhou, China 510120; ^3^Department of Thoracic and Cardiac Surgery, The Eighth Affiliated Hospital, Sun Yat-sen University, Shenzhen, China 518033; ^4^Department of Cardiology, Sun Yat-sen Memorial Hospital, Sun Yat-sen University, Guangzhou, China 510120; ^5^Laboratory of Cardiac Electrophysiology and Arrhythmia in Guangdong Province, Guangzhou, China 510120

## Abstract

Atherosclerosis (AS) is one of the most serious and common cardiovascular diseases affecting human health. AS is featured by the accumulation of plaques in vessel walls. The pathophysiology of AS is relevant in the low-density lipoprotein (LDL) uptake by macrophages, as well as the conversion of macrophages to foam cells. However, the mechanisms about how macrophages regulate AS have not been fully elucidated. In this study, we aimed to illuminate the roles of ZBTB20 and to excavate the underlying regulative mechanisms of ZBTB20 in AS. The microarray analysis revealed that ZBTB20 was a hub gene in the oxidative stress and inflammatory responses induced by oxidized LDL (ox-LDL) in AS. Correspondingly, our validation studies showed that ZBTB20 increased in either the human atherosclerotic lesion or the ox-LDL-stimulated macrophages. Moreover, the knockdown of ZBTB20 decreased M1 polarization, suppressed the proinflammatory factors, inhibited mitochondrial fission, and reduced the oxidative stress level of macrophages induced by ox-LDL. The mechanistic studies revealed that the ZBTB20 knockdown suppressed NF-*κ*B/MAPK activation and attenuated the mitochondrial fission possibly via regulating the nucleus translocation of NRF2, a pivotal transcription factor on redox homeostasis. Our *in vivo* studies showed that the sh-ZBTB20 adenovirus injection could reduce the progression of AS in apolipoprotein E-deficient (ApoE^−/−^) mice. All in all, these results suggested that ZBTB20 positively regulated the oxidative stress level, mitochondrial fission, and inflammatory responses of macrophages induced by ox-LDL, and the knockdown of ZBTB20 could attenuate the development of AS in ApoE^−/−^ mice.

## 1. Introduction

AS is a chronic vascular disease featured by the accumulation of plaques within the vessel wall of the large- and middle-sized arteries [1, 2]. Up to date, various factors including hypertension, diabetes mellitus, high cholesterol level, smoking, and adiposity have been found to be associated with the development of AS [3, 4]. The pathophysiology of AS is closely related to the LDL uptake by macrophages and subsequent differentiation into foam cells. The plaques formed by macrophages have different subtypes based on the activation stimuli and protein expression patterns. Thus, macrophages may exert either harmful effects or beneficial effects in the progression of AS [5, 6]. Recent studies have proposed that targeting of the macrophage may be a crucial target to ameliorate vulnerable plaques and subsequently alleviate AS [7]. However, the underlying mechanistic role of macrophages in the pathophysiology remains unclear. Hence, it is of paramount importance to further elucidate the underlying mechanisms of macrophage-mediated AS, which may provide novel therapies for the treatment.

Recently, studies demonstrated that mitochondria not only can regulate innate immune responses [8] but also can modulate the level of reactive oxygen species (ROS) to affect the homeostasis and inflammatory status of macrophages [9]. There is growing evidence showing that AS may be associated with the dysregulated mitochondrial function and bioenergetics [10]. Chen et al. demonstrated that CD36 signaling regulated mitochondrial metabolic reprogramming, which subsequently drives macrophage inflammatory responses in AS [11]. Besides, Dicer in macrophages was found to prevent AS by promoting mitochondrial oxidative metabolism [12]. Recently, myeloperoxidase-derived oxidant hypothiocyanous acid (HOSCN) was found to induce mitochondrial dysfunction in macrophages, which may be associated with the pathophysiology of AS [13]. Xin et al. also demonstrated that ox-LDL activates the dynamin-related protein 1 (DRP1) level as well as the mitochondrial fission status of macrophages [14].

ZBTB20, mainly known as a transcriptional repressor, is a member of the POZ and Krüppel family, with a zinc finger domain and an intact BTB domain [15, 16]. Up to date, the diverse functions of ZBTB20 have been reported, and studies demonstrated that ZBTB20 could regulate ion channels, remodeling, immunity, and inflammation [17–20]. In vascular diseases, especially in the development of AS, NF-*κ*B signaling functions as a key modulator in AS plaque initiation and evolution [21]. Emerging evidence suggests that the NF-*κ*B pathway was activated during the transformation of cholesterol-rich foam cells after taking in LDL, as well as the oxidation of LDL [22]. As for ZBTB20, in vascular diseases, it can regulate cardiac remodeling after myocardial infarction via ROS/TNF-*α* signaling [23]. Recently, a study by Liu et al. showed that ZBTB20 was able to inhibit the transcription of the *IκBα* gene, which is a key element in NF-*κ*B signaling. Our study has also found that during the macrophage-mediated osteolysis, ZBTB20 could adjust the inflammatory response and polarization of macrophages via regulating *IκBα* transcription and NF-*κ*B activation [18]. However, the roles of macrophage ZBTB20 during the progression of AS remain to be examined.

Here, we examined the expression of ZBTB20 in the macrophages stimulated by ox-LDL and the human AS lesions. Furthermore, the loss-of-function studies were carried out to determine the roles of ZBTB20 on the inflammatory responses, oxidative stress, and mitochondrial fission of the ox-LDL-stimulated macrophages. Besides, the subsequent signaling pathways were also examined. Finally, the effects of ZBTB20 on the AS progression were evaluated in the ApoE^−/−^ mice. In brief, the present study may provide novel insights into the roles of ZBTB20 in the pathophysiology and progression of AS.

## 2. Materials and Methods

### 2.1. Clinical Samples

Clinical samples were collected from 16 patients, including 9 males and 7 females (average age: 57.1 ± 13.1 years). These patients have accepted bypass operation of the coronary artery because of coronary diseases from 2017 to 2019. Those coronary artery tissues containing AS were collected. The internal mammary artery tissues without AS were used as the control group. All procedures were carried out with the approval of the Ethics Committee of Sun Yat-sen University, Sun Yat-sen Memorial Hospital (SYSEC-KY-KS-2020-090).

### 2.2. Cell Lines and Cell Culture

The macrophages, RAW264.7, were from Procell Life Science & Technology. The RAW264.7 macrophages were cultured in high-glucose DMEM, containing 10% fetal bovine serum (FBS) [24]. Cells were cultured at 37°C and 5% CO_2_. The macrophages were seeded 24 h before the experiments.

### 2.3. Small Interfering RNA (siRNA), Cell Transfections, and Treatments

The siRNA targeting ZBTB20 was synthesized by the RiboBio company (Guangzhou, China), and the scrambled siRNA was served as the negative control (NC). For the cell transfections, the ZBTB20-siRNA or the scrambled siRNA was transfected into the macrophages with RNAiMAX (Thermo Fisher Scientific) [25]. Forty-eight hours later, the transfected macrophages were collected for further experimentation.

The ox-LDL was from Yeasen Biotech Co., Ltd., and a concentration of 50 *μ*g/ml was adopted for respective time durations. After that, the macrophages were subjected to further experimental assays. The NRF2 inhibitor (ML385) was purchased from Selleck, and a concentration/duration of 5 *μ*M/24 h was used before further experimental assays [26].

### 2.4. Oil Red O Staining

After treatment, the RAW264.7 macrophages were treated with 4% PFA for 15 min and stained with oil red O solution for 60 min. For the mouse aortas, the adipose tissues were stripped from the aortas [27]. After washing with PBS three times, the aortas were stained with oil red O solution for 60 min. The stained macrophages and tissues were imaged using a biomicroscope (DM2000, Leica).

### 2.5. Dil-ox-LDL Uptake of Macrophages

The macrophages were incubated with red fluorescence-labeled Dil-ox-LDL (50 *μ*g/ml; Yeasen Biotech Co., Ltd.) for 24 h at 37°C [28]. After that, the macrophages were washed by PBS three times, and a biomicroscope (DM2000, Leica) was used.

### 2.6. PPI Network Construction and Identification of Hub Genes

A microarray data of the GSE54666 dataset was obtained from the GEO database. The STRING database and Cytoscape software were used to construct a protein-protein interaction network (PPIN) of differentially expressed genes (DEGs). The topology property of the network was analyzed using the MCODE application of Cytoscape software. The functional clustering of the DEGs was performed using the Metascape online tool (https://metascape.org).

### 2.7. RNA Extraction and qRT-PCR

RNAiso Plus (TaKaRa) was used to collect the RNA of RAW264.7 macrophages. A NanoDrop instrument was used to measure the concentrations of RNA [29]. After that, cDNA was obtained by reverse-transcribing RNA with PrimeScript RT Mix (TaKaRa). Then, qRT-PCR was carried out on a Roche Real-Time PCR System using SYBR Green Mix (Yeasen Biotech Co., Ltd.).  Table 1 shows the primers used.

### 2.8. Western Blot Assay and ELISA

The total protein from RAW264.7 macrophages was obtained by using the RIPA buffer (Beyotime). A nuclear and cytoplasmic extraction kit (CWbiotech) was used to, respectively, obtain the cytosolic protein and nuclear protein. A total of 30 *μ*g protein in each lane was prepared, followed by separating in a 10% polyacrylamide SDS-PAGE gel [30]. After transferring, the PVDF membranes were then blocked with 5% BSA, then incubated with different antibodies including NRF2, Histone H3, p-JNK, KEAP1, DRP1, p-ERK, FIS1, ZBTB20, p-p65, I*κ*B*α*, p-p38, IRF3, p-IRF3, and GAPDH (Cell Signaling Technology) [31]. After the incubation, a secondary antibody with linked HRP (Cell Signaling Technology), an ECL detection kit (Yeasen Biotech Co., Ltd.), and a digital imaging system (Kodak) were used.

To detect the cytokines, including TNF-*α*, IL-6, and IFN-*β*, the macrophage supernatants were collected, followed by detection with ELISA kits (Neobioscience Technology Co., Ltd.).

### 2.9. Flow Cytometry for Macrophage Polarization and ROS Detection

For the detection of macrophage polarization, iNOS and CD206 were adopted as M1 and M2 polarization markers [32]. Briefly, after incubating in the fixation buffer and washing by the perm/wash buffer, the RAW264.7 macrophages were then incubated in the iNOS antibody or CD206 antibody for 20 min, respectively. After that, these macrophages were washed three times, resuspended in 200 *μ*l PBS for each sample, and analyzed with BD Biosciences flow cytometry.

The ROS production was evaluated using a 2′,7′-DHE-DA staining kit (KeyGen Biotech). A concentration of 2 *μ*M and an incubation duration of 20 min were adopted [33]. After the incubation, the ROS-positive cell numbers were detected by BD Biosciences flow cytometry, and the fluorescence intensity of the ROS probe was observed on an Olympus fluorescence microscope.

### 2.10. Mitochondrial Staining of Macrophages

The mitochondrial staining of the macrophages was performed using the MitoTracker Red CMXRos reagent (Beyotime). Briefly, RAW264.7 macrophages after different treatments were incubated with the MitoTracker Red CMXRos reagent for 30 min [34]. Then, the cells were counterstained with DAPI for 10 min and imaged under an Olympus fluorescence microscope.

### 2.11. *In Vivo* Animal Models of Atherosclerosis and Atherosclerotic Lesion Analysis

Adenovirus expressing sh-ZBTB20 or sh-NC was purchased from GeneChem. The ApoE^−/−^ mice (20-30 grams, 8-10 weeks) were purchased from GemPharmatech Co. Ltd. To study the effects of the ZBTB20 knockdown on the AS mouse model, the animals were injected with adenovirus expressing sh-ZBTB20 or sh-NC via the tail, and then the ApoE^−/−^ mice were fed with a high-fat diet for 12 weeks. At the end of the experiments [35], the animals were killed by an overdose of pentobarbitone (80 mg/kg, intravenous injection), and the tissues were collected for further experimental assays. All the guidelines of the Institutional Animal Care and Use Committee of Sun Yat-sen University were followed during the animal experiments [36].

The collected tissues were fixed and embedded, then sliced into sections using a microtome. The sections were then dewaxed using the xylene. The H&E staining kit (Beyotime), CD68 antibody (Servicebio), Masson stain kit (Solarbio), *α*-SMA antibody (Beyotime), and EVG stain kit (Abcam) were used to evaluate the AS lesions [37].

### 2.12. Immunofluorescent Staining of Macrophages

For the immunofluorescent staining of the macrophages, the macrophages with different treatments were fixed with 4% PFA, followed by incubation with 0.1% Triton X-100 and blocking with 1% BSA. Then, the macrophages were incubated with TNF-*α*, IL-6, p65, IRF3, or NRF2 antibodies (Cell Signaling Technology) overnight at 4°C [38]. Alexa Fluor 555 conjugate immunofluorescent secondary antibodies and a Carl Zeiss confocal microscope were used to observe the macrophages.

### 2.13. Statistical Analysis

Data are presented as mean ± standard deviation. The data normality was analyzed by the Kolmogorov–Smirnov test. Two-sided Student's *t*-test and one-way analysis of variance followed by Fisher's least significant difference test were performed with the SPSS 20.0 software. The differences between means were considered significant when *P* < 0.05.

## 3. Results

### 3.1. TNF-*α* and IL-6 Were Upregulated in the AS Lesions and the ox-LDL-Stimulated Macrophages

**Figure 1:**
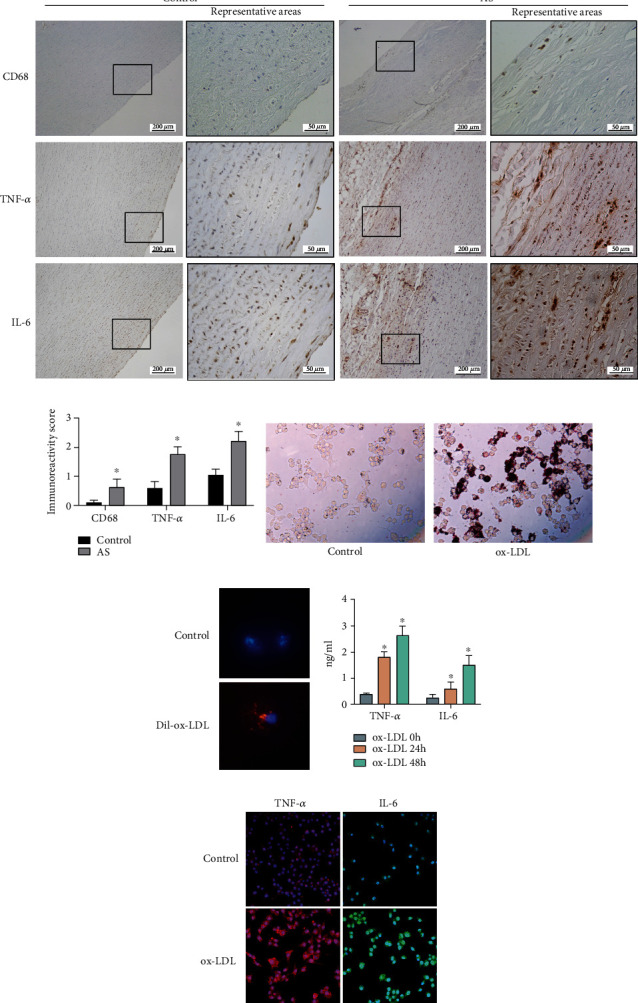
TNF-*α* and IL-6 were upregulated in the atherosclerotic aorta and the ox-LDL-stimulated macrophages. (a) The protein expression of CD68, TNF-*α*, and IL-6 in the normal aorta and atherosclerotic aorta was determined by immunofluorescent staining. (b) The immunostaining scores in the normal aorta and atherosclerotic aorta were determined. (c) The uptake of ox-LDL by macrophages was assessed by oil red O staining. (d) The uptake of Dil-ox-LDL by macrophages was assessed by immunofluorescent staining. (e) The TNF-*α* and IL-6 cytokines secreted by macrophages after being treated with ox-LDL for 0, 24, and 48 h, respectively, were determined by ELISA. (f) The TNF-*α* and IL-6 protein expression levels in the macrophages after being treated with ox-LDL for 24 h were determined by immunofluorescent staining. *N* = 3; significant differences between treatment groups were shown as ^∗^*P* < 0.05.

Firstly, we examined the expression levels of CD68, TNF-*α*, and IL-6 in the lesion area from the patients with AS by IHC staining. The protein expression of CD68, TNF-*α*, and IL-6 was significantly upregulated in the lesion area from the patients with AS compared to the control group (Figures 1(a) and 1(b)). We further treated the macrophages with ox-LDL for 24 h, and the differentiation of macrophages into foam cells was observed by oil red O staining (Figure 1(c)). In addition, the fluorescence microscope showed that the macrophages could directly take in Dil-ox-LDL, which was red fluorescence-labeled (Figure 1(d)). The ELISA results showed that inflammatory cytokines were also significantly elevated in the macrophages induced by ox-LDL for 24 h and 48 h (Figure 1(e)). In addition, the mRNA levels of TNF-*α* and IL-6 were upregulated and induced by ox-LDL (Fig. S1A). Consistently, the fluorescent signaling intensities of these two inflammatory proteins significantly increased in macrophages because of the treatment of ox-LDL (Figure 1(f)).

### 3.2. ox-LDL Increased the Oxidative Stress and Mitochondrial Injury via Modulating NRF2

**Figure 2:**
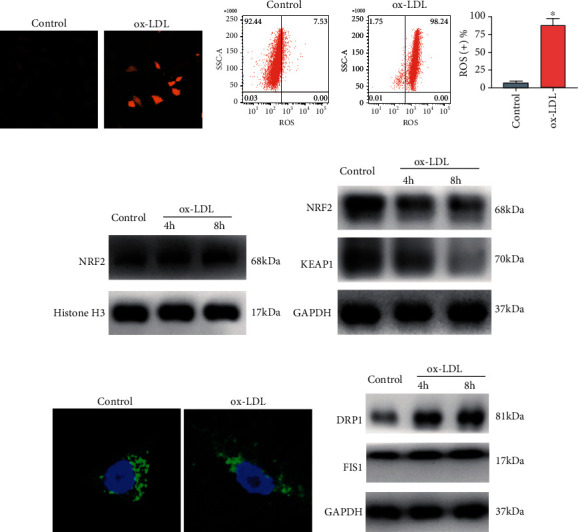
ox-LDL increased the oxidative stress and mitochondrial injury via modulating NRF2. (a) The ROS level of the PBS- or ox-LDL-treated macrophages was determined by immunofluorescent staining. (b) The ROS-positive rates of the PBS- or ox-LDL-treated macrophages with different treatments were determined by flow cytometry. (c) The protein level of NRF2 in the nucleus of the PBS- or ox-LDL-treated macrophages was assessed by Western blot assay. (d) The protein level of NRF2 and KEAP1 in the cytoplasm of the PBS- or ox-LDL-treated macrophages was assessed by Western blot assay. (e) The mitochondrial injury of the PBS- or ox-LDL-treated (6 h) macrophages was assessed by MitoTracker staining. (f) The protein level of DRP1 and FIS1 in the PBS- or ox-LDL-treated (6 h) macrophages with respective treatments was assessed by Western blot assay. *N* = 3; significant differences between treatment groups were shown as ^∗^*P* < 0.05.

Here, ox-LDL treatment significantly enhanced the ROS production in the macrophages and also raised the number of ROS-positive macrophages (Figures 2(a) and 2(b) and Fig. S1B). The Western blot results showed that ox-LDL stimulation significantly raised the protein level of NRF2 in the nucleus of macrophages but decreased NRF2 and KEAP1 protein levels in the cytoplasm of the macrophages (Figures 2(c) and 2(d) and Fig. S1C-S1D). Moreover, the MitoTracker staining showed that ox-LDL stimulation significantly induced the mitochondrial injury of the macrophages (Figure 2(e)). Besides, the ox-LDL stimulation for 4 and 8 h both increased the protein levels of DRP1 and FIS1 in the macrophages (Figure 2(f) and Fig. S1E).

### 3.3. ZBTB20 Was Identified as the Hub Gene Associated with Oxidative Stress and Inflammation in ox-LDL-Stimulated Macrophages

**Figure 3:**
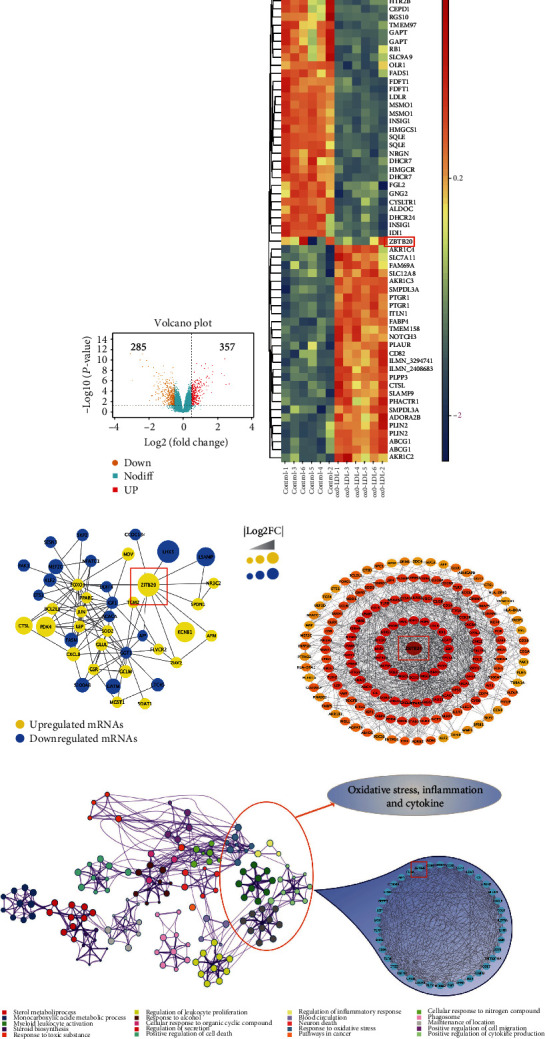
ZBTB20 was identified as the hub gene associated with oxidative stress, inflammation, and cytokines in the ox-LDL-stimulated macrophages. (a) Volcano plots and (b) heat map illustration of the differentially expressed genes between control macrophages and ox-LDL-stimulated macrophages. (c) The hub genes from the PPI network were further identified using the MCODE app from Cytoscape software. (d) The cytoHubba plug-in from Cytoscape software was used, and the hub gene network was obtained according to the core gene scores. (e) The functional clustering of genes associated with oxidative stress, inflammation, and cytokines was performed using the Metascape database.

The DEGs between the treatment group and the control group from the GSE54666 dataset were illustrated in the volcano plot and heat map, respectively (Figures 3(a) and 3(b)). A total of 642 DEGs were identified, including 357 upregulated DEGs and 285 downregulated DEGs. The DEGs were then subjected to the PPI network construction by using the STRING database, and a total of 538 nodes and 2037 edges were identified in the PPI network. The top 15 significant GO terms and KEGG pathways from the GO categories and KEGG database were shown in Fig. S2A, and the four most significant KEGG pathways analyzed with GSEA were shown in Fig. S2B. Furthermore, the hub genes were extracted using the MCODE application in the Cytoscape software. Eighty-six hub genes were identified, and the ZBTB20 gene had a high score among them (Figure 3(c)). Besides, the hub gene network was obtained according to the core gene scores by the cytoHubba plug-in (Figure 3(d)). The functional clustering of the hub genes showed that ZBTB20 was associated with oxidative stress, inflammation, and cytokines (Figure 3(e)).

### 3.4. ZBTB20 Promoted the Inflammatory Responses of the ox-LDL-Stimulated Macrophages

**Figure 4:**
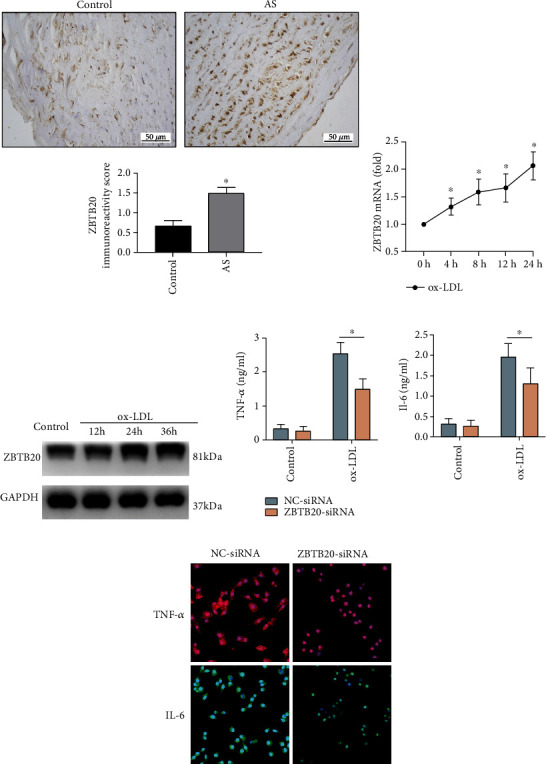
ZBTB20 promotes the inflammatory responses of the ox-LDL-stimulated macrophages. (a) The protein expression of ZBTB20 in the normal aorta and atherosclerotic aorta was determined by immunofluorescent staining. The (b) mRNA and (c) protein expression levels of ZBTB20 in the macrophages after being treated with ox-LDL for 0, 4, 8, or 24 h were determined by qRT-PCR and Western blot assays, respectively. (d) The TNF-*α* and IL-6 protein expression levels in the PBS- or ox-LDL-treated macrophages after being transfected with scrambled siRNA (NC) or si-ZBTB20 were determined by ELISA. (e) The TNF-*α* and IL-6 protein expression levels in the ox-LDL-treated macrophages after being transfected with scrambled siRNA (NC) or si-ZBTB20 were evaluated by immunofluorescent staining. *N* = 3; significant differences between treatment groups were shown as ^∗^*P* < 0.05.

The expression of ZBTB20 as illustrated by the IHC staining was significantly upregulated in the lesion area from the patients with AS compared to the control group (Figure 4(a)). Western blot as well as qRT-PCR showed that ox-LDL time-dependently increased the expression of ZBTB20 in the macrophages not only in the mRNA level but also in the protein level (Figures 4(b) and 4(c) and Fig. S3A). The Western blot result of ZBTB20-siRNA knockdown efficiency was shown in Fig. S3B. The ELISA results showed that the knockdown of ZBTB20 significantly reduced the inflammatory cytokine levels secreted by ox-LDL-induced macrophages (Figure 4(d)). Consistently, the ZBTB20 knockdown significantly reduced the fluorescent signaling intensities of TNF-*α* and IL-6 proteins in ox-LDL-induced macrophages (Figure 4(e)).

### 3.5. ZBTB20 Knockdown Suppressed the NK-*κ*B and MAPK Signaling Activities in the ox-LDL-Stimulated Macrophages

**Figure 5:**
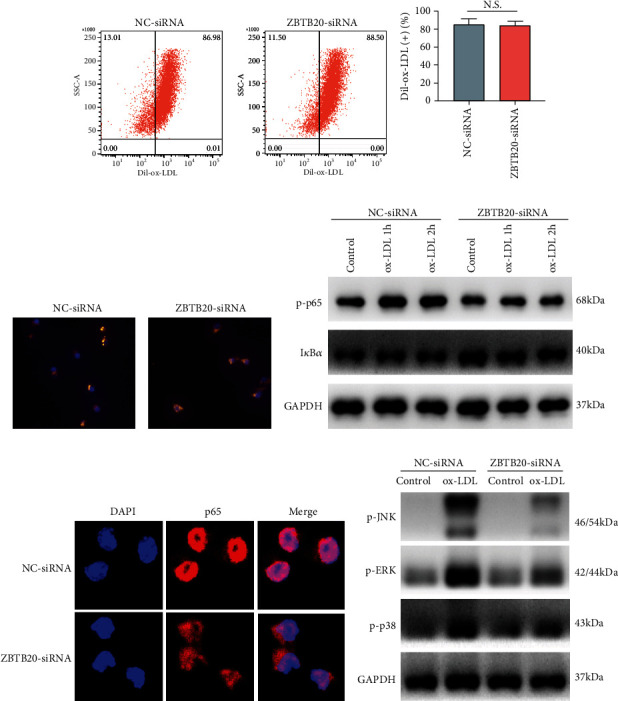
ZBTB20 knockdown suppresses the NF-κB and MAPK signaling activities in the ox-LDL-stimulated macrophages. (a, b) The ox-LDL uptake of macrophages after being transfected with scrambled siRNA (NC) or si-ZBTB20 was determined by flow cytometry. (c) The ox-LDL uptake of macrophages after being transfected with scrambled siRNA (NC) or si-ZBTB20 was determined by immunofluorescent staining. (d) The protein expression levels of the NF-κB-related mediators in the PBS- or ox-LDL-treated macrophages after being transfected with scrambled siRNA (NC) or si-ZBTB20 were determined by Western blot assay. (e) The protein expression level of p65 in ox-LDL-treated (1 h) macrophages after being transfected with scrambled siRNA (NC) or si-ZBTB20 was determined by immunofluorescent staining. (f) The protein expression levels of the MAPK-related mediators in the PBS- or ox-LDL-treated (1 h) macrophages after being transfected with scrambled siRNA (NC) or si-ZBTB20 were determined by Western blot assay. *N* = 3.

Here, the effects of the ZBTB20 knockdown on the ox-LDL uptake of macrophages were determined by fluorescent staining and flow cytometry. As shown in Figures 5(a) and 5(b) and Fig. S3C, the ZBTB20 knockdown had no significant effects on the Dil-ox-LDL-positive macrophage ratios as determined by flow cytometry. Consistently, there was no significant difference in the fluorescent signaling intensity of Dil-ox-LDL in the macrophages between the NC-siRNA and ZBTB20-siRNA groups as determined by flow cytometry (Figure 5(c)).

The effects of the ZBTB20 knockdown on the protein levels of the NK-*κ*B-related mediators in the macrophages were determined by the Western blot assay. Interestingly, the ZBTB20 knockdown significantly reduced the protein level of p-p65 but increased that of I*κ*B*α* in the ox-LDL-stimulated macrophages when compared to the PBS-treated macrophages (Figure 5(d) and Fig. S3D). Consistently, the fluorescent staining showed that the ZBTB20 knockdown suppressed the nucleus translocation of p65 in the ox-LDL-stimulated macrophages (Figure 5(e)). In addition, the MAPK-related mediators were also examined, and as shown in Figure 5(f) and Fig. S3E, the ZBTB20 knockdown significantly downregulated the phosphorylation levels of JNK, ERK, and p38 induced by ox-LDL in macrophages.

### 3.6. ZBTB20 Knockdown Suppressed M1 Polarization, Increased M2 Polarization, and Inhibited the Phosphorylation and Nucleus Translocation of IRF3

**Figure 6:**
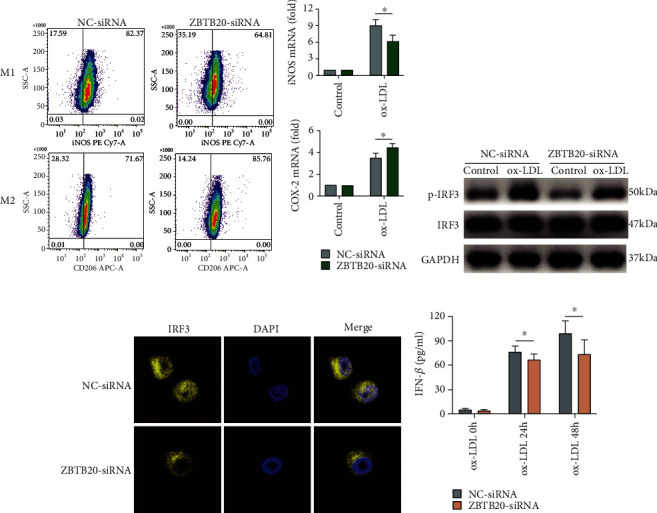
ZBTB20 knockdown suppresses the M1 polarization, increases the M2 polarization, and inhibits the phosphorylation and nucleus translocation of IRF3 in the ox-LDL-stimulated macrophages. (a) The M1 and M2 polarization of the ox-LDL-treated (24 h) macrophages after being transfected with scrambled siRNA (NC) or si-ZBTB20 was determined by flow cytometry. (b) The mRNA expression level of the iNOS in the PBS- or ox-LDL-treated (24 h) macrophages after being transfected with scrambled siRNA (NC) or si-ZBTB20 was determined by qRT-PCR. (c) The phosphorylation level of the IRF3 in the PBS- or ox-LDL-treated (8 h) macrophages after being transfected with scrambled siRNA (NC) or si-ZBTB20 was determined by Western blot assay. (d) The nucleus translocation of IRF3 in the ox-LDL-treated (8 h) macrophages after being transfected with scrambled siRNA (NC) or si-ZBTB20 was determined by immunofluorescent staining. (e) The cytokine level of IFN-*β* in the ox-LDL-treated macrophages after being transfected with scrambled siRNA (NC) or si-ZBTB20 was determined by ELISA. *N* = 3; significant differences between treatment groups were shown as ^∗^*P* < 0.05.

Here, the effects of the ZBTB20 knockdown on M1/M2 polarization of macrophages were determined by flow cytometry, and as presented in Figure 6(a) and Fig. S4A-S4B, the percentage of macrophages with M1 polarization significantly decreased, and the percentage of macrophages with M2 polarization increased in the ZBTB20-siRNA group compared to the NC-siRNA group. The qRT-PCR results showed that the ZBTB20 knockdown downregulated the mRNA level of iNOS, an M1 polarization marker, but upregulated the mRNA level of COX-2, an M2 polarization marker, in the ox-LDL-stimulated macrophages (Figure 6(b)). Interestingly, the Western blot results showed that the ZBTB20 knockdown significantly suppressed the phosphorylation of the IRF3 in the ox-LDL-stimulated macrophages, but not in the PBS-treated macrophages (Figure 6(c) and Fig. S4C). In addition, the fluorescent staining results showed that the ZBTB20 knockdown repressed the nucleus translation of IRF3 in the ox-LDL-stimulated macrophages (Figure 6(d)). ELISA showed that the ZBTB20 knockdown reduced the cytokine levels of IFN-*β* in the ox-LDL-stimulated macrophages (Figure 6(e)).

### 3.7. ZBTB20 Regulated Oxidative Stress and Mitochondrial Fission in ox-LDL-Stimulated Macrophages via Modulating NRF2

**Figure 7:**
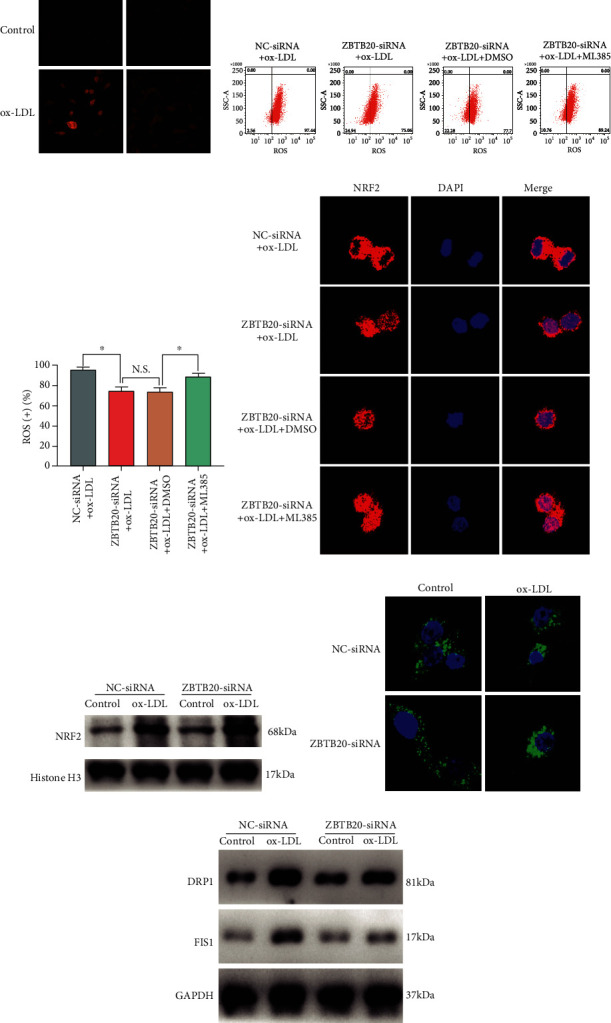
ZBTB20 positively regulates oxidative stress and mitochondrial fission in ox-LDL-stimulated macrophages via modulating NRF2. (a) The ROS level in the PBS- or ox-LDL-treated macrophages after being transfected with scrambled siRNA (NC) or si-ZBTB20 was determined by immunofluorescent staining. (b, c) The ROS-positive rates of the macrophages with different treatments were determined by flow cytometry. (d) The nucleus translation of NRF2 in the ox-LDL-stimulated macrophages with respective treatments was determined by immunofluorescent staining. (e) The protein level of NRF2 in the nucleus of the macrophages with respective treatments was assessed by Western blot assay. (f) The mitochondrial injury of the macrophages with respective treatments was assessed by MitoTracker staining. (g) The protein level of DRP1 and FIS1 in the macrophages with respective treatments was assessed by Western blot assay. *N* = 3; significant differences between treatment groups were shown as ^∗^*P* < 0.05.

The ROS production in the macrophages with respective treatments was analyzed using fluorescent staining and flow cytometry. The fluorescent staining results showed that the treatment of ox-LDL significantly increased the ROS level in the macrophages, which was significantly attenuated by the ZBTB20 knockdown (Figure 7(a)). Consistently, the ZBTB20 knockdown attenuated the ox-LDL-induced increase of ROS-positive macrophages, and more importantly, the effects of the ZBTB20 knockdown on the number of ROS-positive macrophages were antagonized by the treatment of ML385, a novel NRF2 inhibitor (Figures 7(b) and 7(c) and Fig. S4D). The fluorescent staining results showed that the knockdown of ZBTB20 enhanced the nucleus translocation of NRF2 induced by ox-LDL, which was also attenuated by ML385 treatment (Figure 7(d)). Consistently, the ZBTB20 knockdown increased the nuclear expression level of NRF2 protein in ox-LDL-stimulated macrophages as determined by the Western blot assay (Figure 7(e) and Fig. S4E). Furthermore, MitoTracker staining showed that the ZBTB20 knockdown prevented the mitochondrial injury in the macrophages (Figure 7(f)), and the increased protein expression levels of DRP1 and FIS1 induced by ox-LDL were significantly attenuated by the ZBTB20 knockdown as well (Figure 7(g) and Fig. S4F-S4G).

### 3.8. Knockdown of ZBTB20 Attenuates the Development of AS in ApoE^−/−^ Mice

**Figure 8:**
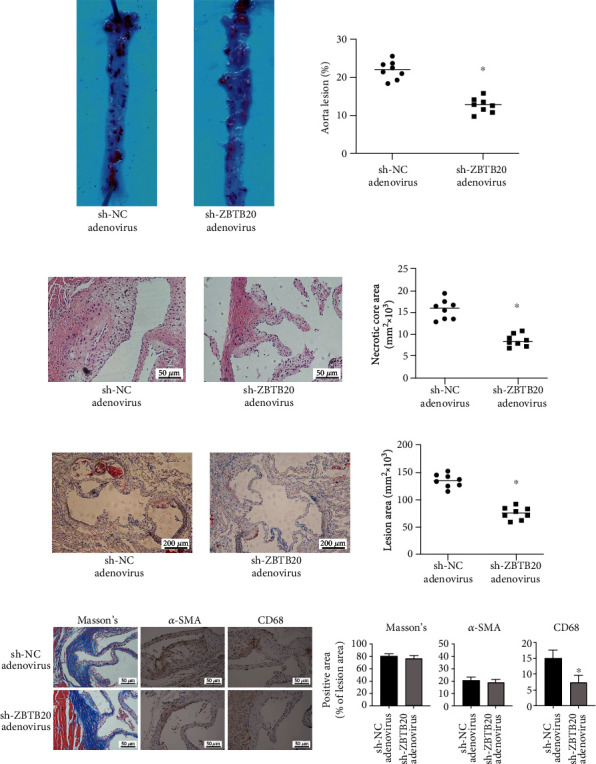
Knockdown of ZBTB20 attenuates the progression of the atherosclerosis of the ApoE^−/−^ mice. (a) Representative images of the oil red O staining in whole aortas from sh-NC-treated ApoE^−/−^ mice or sh-ZBTB20-treated ApoE^−/−^ mice fed with a high-fat diet for 16 weeks. ImageJ software was used to quantify the lesion coverage of the entire aorta (%). (b) Representative images of H&E staining in cross-sections of the aortic root from mice in different groups. ImageJ software was used to quantify areas of the necrotic core. (c) Representative images of oil red O staining in cross-sections of the aortic root from mice in different groups. ImageJ software was used to assess the lesion areas. (d) Representative images of Masson staining for collagen content, IHC staining for SMC content, and macrophage accumulation (CD68) in whole aortas from sh-NC-treated ApoE^−/−^ mice or sh-ZBTB20-treated ApoE^−/−^ mice fed with a high-fat diet for 16 weeks. *N* = 8; significant differences between treatment groups were shown as ^∗^*P* < 0.05.

The effects of the ZBTB20 knockdown on the progression of AS were evaluated in ApoE^−/−^ mice, which were fed with a high-fat diet for 12 weeks. As shown in Figure 8(a), the tail vein injection of the adenovirus expressing sh-ZBTB20 significantly reduced the aorta lesion in the ApoE^−/−^ mice when compared to animals treated with control adenovirus. The H&E staining showed consistent results (Figure 8(b)). Furthermore, the foam cells in the aorta were stained by the oil red, and the lesion area significantly decreased in the sh-ZBTB20 group (Figure 8(c)). Besides, we performed Masson staining for collagen content, IHC staining for smooth muscle cell (SMC) content (*α*-SMA (*α*-smooth muscle actin)), and macrophage accumulation (CD68) in whole aortas from sh-NC-treated ApoE^−/−^ mice and sh-ZBTB20-treated ApoE^−/−^ mice (Figure 8(d)), and the results showed that the knockdown of ZBTB20 attenuated the macrophage accumulation in the progression of AS but had no effects on collagen or SMC content.

## 4. Discussion

AS progression is closely related to proinflammatory and proatherogenic mediators, which can promote plaque formation and stenosis progression [39]. In the initiation of AS, high levels of ox-LDL can recruit monocytes, promoting the adhesion molecule expression on the endothelium and the subsequent adhesion of the monocytes to the intima [40]. Here, our microarray analysis revealed that ZBTB20 was identified as the hub gene in the ox-LDL-stimulated macrophages. The validation studies showed that ZBTB20 was upregulated in the human AS lesions and ox-LDL-stimulated macrophages. The loss-of-function studies showed that the ZBTB20 knockdown suppressed the proinflammatory cytokine levels, decreased the M1 polarization, and reduced the oxidative stress and mitochondrial fission in the ox-LDL-stimulated macrophages. The mechanistic studies showed that the ZBTB20 knockdown not only suppressed the NK-*κ*B and MAPK signaling activities but also inhibited the nucleus translocation of NRF2 in the ox-LDL-stimulated macrophages. Our *in vivo* data showed that the ZBTB20 knockdown attenuated the development of AS in ApoE^−/−^ mice.

The present study showed the increased expression levels of proinflammatory factors in the human AS lesions and ox-LDL-stimulated macrophages, which was consistent with previous studies [41, 42]. These results indicated the increased inflammatory response in the macrophages during AS. The upregulation of ZBTB20 in human AS lesions and ox-LDL-stimulated macrophages suggested that ZBTB20 may promote the initiation and progression of AS. Thus, we performed the loss-of-function studies by silencing the ZBTB20 gene. In this study, the knockdown of ZBTB20 suppressed proinflammatory protein levels in ox-LDL-induced macrophages, which was consistent with studies by Qiu et al., showing that ZBTB20 silencing suppressed the inflammatory responses in titanium particle-stimulated or lipopolysaccharide- (LPS-) stimulated macrophages [18]. In addition, the ZBTB20 knockout was reported to decrease the serum levels of IL-6 and TNF-*α* in LPS-treated mice [20]. AS is an inflammatory disease, and NF-*κ*B functions as a major transcription factor in inflammatory and immune responses [43, 44]. Thus, we further examined the roles of the ZBTB20 knockdown on NF-*κ*B activities, and we found that the ox-LDL stimulation increased the activities of macrophage NF-*κ*B signaling, which was consistent with findings from previous studies [45, 46]. ZBTB20 was also found to promote the activity of NF-*κ*B in the gastric cancer cells and human dental pulp stem cells [47, 48]. Besides, the present study showed that ox-LDL enhanced the activity of MAPK, another inflammatory signaling, in macrophages, which was consistent with previous reports from Taketa et al. [49]. Here, our further results revealed that the ZBTB20 knockdown reduced MAPK activity in ox-LDL-stimulated macrophages. However, studies from Liu et al. showed that ZBTB20 had no effects on Toll-like receptor-triggered activation of MAPK [20]. The inconsistent effects of ZBTB20 on the activity of MAPK in macrophages among different studies might be attributed to the different stimuli used. Collectively, in this study, the inhibitory effects of the ZBTB20 knockdown on the inflammatory responses in the macrophages may be related to the impaired activity of NF-*κ*B and MAPK.

Macrophages can be divided into M1 or M2 type, depending on the polarization state, all of which were derived from monocytes [50]. Recent studies showed that M2 macrophages could clear dying cells and debris and secrete anti-inflammatory factors, which can attenuate the formation of AS plaques [51]. Our results showed that the ZBTB20 knockdown increased the M2 macrophage polarization but decreased the M1 macrophage polarization, implying that the ZBTB20 knockdown may attenuate AS by activating M2 macrophages. IRF3 is a key interferon-regulator factor in regulating the M2 polarization of macrophages, and IRF3 can cooperate with NF-*κ*B to launch IFN-*β* gene transcription [52]. Consistently, our data showed that the ZBTB20 knockdown suppressed the phosphorylation and nucleus translocation of IRF3 and reduced the IFN-*β* cytokine level in the ox-LDL-stimulated macrophages, suggesting that ZBTB20 regulated the phenotype switching of macrophages by targeting IRF3 and regulating IFN-*β*. Similarly, in the clinic, it may be possible to use macrophage polarization as a target to treat macrophage-related inflammatory diseases, such as aseptic loosening and immune rejection.

It has been widely reported that ox-LDL induced oxidative stress in macrophages [53, 54], and our results consistently elucidated that ox-LDL increased the ROS production and DRP1/FIS1 protein. NRF2 is a crucial transcription factor that regulates oxidative stress responses, and it is required for the antioxidant responses in macrophages [47]. Our results showed that the ZBTB20 knockdown attenuated the ox-LDL-induced ROS production and increased the nucleus translocation of NRF2 in the macrophages, which was significantly antagonized by the NRF2 inhibitor ML385. In addition, the ZBTB20 knockdown also rescued the ox-LDL-induced mitochondrial injury in the macrophages. Collectively, these results suggested that the ZBTB20 knockdown exerted antioxidative stress effects via enhancing the nucleus translocation of NRF2 and preventing the mitochondrial injury in macrophages. Some previous studies suggested that ZBTB20 ablation could protect mice from liver steatosis and improve hepatic lipid metabolism, the dysregulation of which may lead to AS [55]. Here, our *in vivo* data showed that the knockdown of ZBTB20 attenuated AS in ApoE^−/−^ mice. Hence, it is possible to selectively target ZBTB20 using novel viral vectors, such as adenovirus and adeno-associated virus, to slow the progression of clinical immune-inflammatory diseases. However, the *in vivo* effects of ZBTB20 on the AS progression, as well as how ZBTB20 regulates other cell types in AS, such as endotheliocytes, still require further investigations.

Mitochondria are well known for their roles in integrating redox, efferocytosis, epigenetic, and apoptotic regulations [56]. How mitochondria function will depend on the shape and density, which are modulated by the fusion/fission balances [57, 58]. These functions have been found to be disturbed in macrophages from the AS plaques [59]. In this study, we found that ox-LDL stimulation caused ROS production and mitochondrial injury, which were significantly attenuated by ZBTB20 silencing. Our findings were consistent with studies from Peng et al., showing that ox-LDL induced mitophagy in mitochondria [60]. DRP1 and FIS1 are two key mediators relevant to the mitochondrial fission process [61, 62], and our results showed that the ZBTB20 knockdown attenuated the ox-LDL-induced activation of DRP1/FIS1 protein. Besides, our results showed that the knockdown of ZBTB20 enhanced the nucleus translocation of NRF2 in the ox-LDL-stimulated macrophages, which was counteracted by ML385, a novel NRF2 inhibitor. Nevertheless, the NRF2 transcription factor might be one of the signaling pathways through which ZBTB20 regulated the oxidative stress level, and the deeper mechanisms remain to be explored.

Collectively, our results may imply that ZBTB20 silencing reduced oxidative stress by reducing ROS production and mitochondrial injury via NRF2 signaling. In the clinic, it may be possible to use adenovirus or adeno-associated virus targeting ZBTB20 to prevent and treat AS.

## 5. Conclusions

In conclusion, this study demonstrated that the ZBTB20 knockdown could attenuate the progression of AS, and ZBTB20 mediated ox-LDL-induced AS possibly via modulating the inflammatory responses, oxidative stress, and mitochondrial fission of macrophages.

## Figures and Tables

**Table 1:** Primers used in this study.

ZBTB20	Forward	GTGGACCGAATCTACTCCGC
	Reverse	CATGAATGCGTGTGATCCAGC

iNOS	Forward	GGAGTGACGGCAAACATGACT
	Reverse	TCGATGCACAACTGGGTGAAC

COX-2	Forward	TGCACTATGGTTACAAAAGCTGG
	Reverse	TCAGGAAGCTCCTTATTTCCCTT

GAPDH	Forward	TGTGTCCGTCGTGGATCTGA
	Reverse	TTGCTGTTGAAGTCGCAGGAG

## Data Availability

All the datasets were available from the corresponding authors.

## Supplementary Materials

Supplementary Materials Fig. S1: (A) The mRNA levels of TNF-*α* and IL-6 after being treated with ox-LDL for 0, 12, 24, and 48 h were determined by qRT-PCR. (B) The blank control of Figure 2(b). (C) Quantitative analysis of Western blot results in Figure 2(c). (D) Quantitative analysis of Western blot results in Figure 2(d). (E) Quantitative analysis of Western blot results in Figure 2(f). Significant differences between treatment groups were shown as ^∗^*P* < 0.05. Fig. S2: (A) The top 15 significant GO terms and KEGG pathways from the GO categories and KEGG database. (B) The four most significant KEGG pathways analyzed with GSEA. Fig. S3: (A) Quantitative analysis of Western blot results in Figure 4(c). (B) The Western blot result of ZBTB20-siRNA knockdown efficiency. (C) The blank control of Figure 5(a). (D) Quantitative analysis of Western blot results in Figure 5(d). (E) Quantitative analysis of Western blot in Figure 5(f). Significant differences between treatment groups were shown as ^∗^*P* < 0.05. Fig. S4: (A) The isotype controls of Figure 6(a). (B) Quantitative analysis of flow cytometry results in Figure 6(a). (C) Quantitative analysis of Western blot results in Figure 6(c). (D) The blank control of Figure 7(b). (E) Quantitative analysis of Western blot results in Figure 7(e). (F, G) Quantitative analysis of Western blot results in Figure 7(g). Significant differences between treatment groups were shown as ^∗^*P* < 0.05.
