# Supplementary material for: ZBTB20 Positively Regulates Oxidative Stress, Mitochondrial Fission, and Inflammatory Responses of ox-LDL-Induced Macrophages in Atherosclerosis
Source: Oxid Med Cell Longev. 2021 Mar 9;2021:5590855. doi: 10.1155/2021/5590855 (PMC7972849; doi:10.1155/2021/5590855)
Supplement: Supplementary Materials — Fig. S1: (A) The mRNA levels of TNF-α and IL-6 after being treated with ox-LDL for 0, 12, 24, and 48 h were determined by qRT-PCR. (B) The blank control of Figure 2(b). (C) Quantitative analysis of Western blot results in Figure 2(c). (D) Quantitative analysis of Western blot results in Figure 2(d). (E) Quantitative analysis of Western blot results in Figure 2(f). Significant differences between treatment groups were shown as ∗P < 0.05. Fig. S2: (A) The top 15 significant GO terms and KEGG pathways from the GO categories and KEGG database. (B) The four most significant KEGG pathways analyzed with GSEA. Fig. S3: (A) Quantitative analysis of Western blot results in Figure 4(c). (B) The Western blot result of ZBTB20-siRNA knockdown efficiency. (C) The blank control of Figure 5(a). (D) Quantitative analysis of Western blot results in Figure 5(d). (E) Quantitative analysis of Western blot in Figure 5(f). Significant differences between treatment groups were shown as ∗P < 0.05. Fig. S4: (A) The isotype controls of Figure 6(a). (B) Quantitative analysis of flow cytometry results in Figure 6(a). (C) Quantitative analysis of Western blot results in Figure 6(c). (D) The blank control of Figure 7(b). (E) Quantitative analysis of Western blot results in Figure 7(e). (F, G) Quantitative analysis of Western blot results in Figure 7(g). Significant differences between treatment groups were shown as ∗P < 0.05. [file 5590855.f1.zip › S1 (2).pdf]

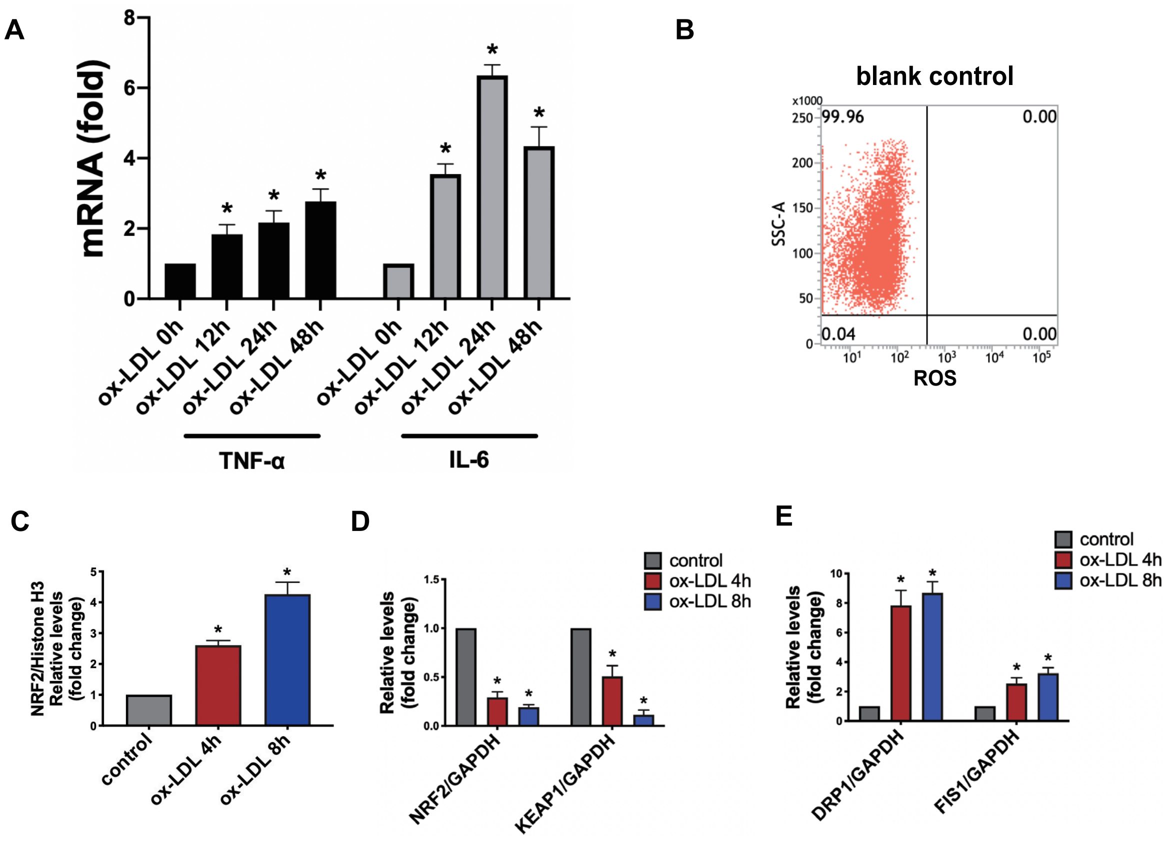

Fig. S1. (A) The mRNA levels of TNF- α and IL-6 after being treated with ox-LDL for 0, 12, 24 and 48 h were determined by qRT-PCR. (B) The blank control of Fig. 2B. (C) Quantitative analysis of Western blot results in Fig. 2C. (D) Quantitative analysis of Western blot results in Fig. 2D. (E) Quantitative analysis of Western blot results in Fig. 2F. Significant differences between treatment groups were shown as *P < 0.05.
